# Timing of Locomotor Activity Circadian Rhythms in *Caenorhabditis elegans*

**DOI:** 10.1371/journal.pone.0007571

**Published:** 2009-10-27

**Authors:** Sergio H. Simonetta, María Laura Migliori, Andrés Romanowski, Diego A. Golombek

**Affiliations:** Departamento de Ciencia y Tecnología, Universidad Nacional de Quilmes and National Research Council (CONICET), Buenos Aires, Argentina; Vanderbilt University, United States of America

## Abstract

Circadian rhythms are driven by endogenous biological clocks and are synchronized to environmental cues. The chronobiological study of *Caenorhabditis elegans*, an extensively used animal model for developmental and genetic research, might provide fundamental information about the basis of circadian rhythmicity in eukaryotes, due to its ease of use and manipulations, as well as availability of genetic data and mutant strains. The aim of this study is to fully characterize the circadian rhythm of locomotor activity in *C. elegans*, as well as a means for genetic screening in this nematode and the identification of circadian mutants. We have developed an infrared method to measure locomotor activity in *C. elegans* and found that, under constant conditions, although inter-individual variability is present, circadian periodicity shows a population distribution of periods centered at 23.9±0.4 h and is temperature-compensated. Locomotor activity is entrainable by light-dark cycles and by low-amplitude temperature cycles, peaking around the night-day transition and day, respectively. In addition, lin-42(mg152) or lin-42(n1089) mutants (bearing a mutation in the *lin-42* gene, homolog to the *per* gene) exhibit a significantly longer circadian period of 25.2±0.4 h or 25.6±0.5 h, respectively. Our results represent a complete description of the locomotor activity rhythm in *C. elegans*, with a methodology that allowed us to uncover three of the key features of circadian systems: entrainment, free-running and temperature compensation. In addition, abnormal circadian periods in clock mutants suggest a common molecular machinery responsible for circadian rhythmicity. Our analysis of circadian rhythmicity in *C. elegans* opens the possibility for further screening for circadian mutations in this species.

## Introduction

Circadian rhythms of gene expression and behavior are ubiquitous in nature. They are generated by endogenous clocks that are entrained by environmental cues, such as light or temperature, and have been described in several animal models. Indeed, the use of the adequate model systems accelerates research and might facilitate the search for the genetic basis of behavior and disease. The molecular central pacemaker consists of a series of feedback loops that regulate the expression of specific clock genes (such as *period* or *timeless*), as well as post-translational events that finely tune the dynamics of the cycle. Diverse chronobiological models have contributed to the understanding of the properties of the circadian system and the molecular machinery of the biological clock, emphasizing common mechanisms in organisms as diverse as fungi, flies and mammals [1], [2].

In this sense, the chronobiological study of *Caenorhabditis elegans*, an extensively used animal model for developmental and genetic research, might provide fundamental information about the basis of circadian rhythmicity in eukaryotes, due to its ease of use and manipulations, as well as availability of genetic data and mutant strains. Interestingly, not much is known about circadian behaviors or putative clock genes in this nematode. In particular, rhythms in swimming behavior [3] and response to osmotic stress [4], [5] have been reported in L1 larvae and adults, as well as preliminary results in locomotor activity of adults [6], while several putative homologs to clock genes that have been characterized in other systems have also been proposed [7], [8]. However, several of these genes have been described as being involved in other regulatory mechanisms, such as developmental processes, including lin-42 (a *period* homolog) that is expressed during molting in several cells [9], or a *timeless* homolog which appears to be involved in chromosomal cohesion [10], [11]. The relationship of these genes to circadian rhythmicity is currently unknown.

On the other hand, ultradian rhythms (i.e., with a period shorter than 20 h, including cycles in the second-to-hours range) have been extensively described in *C. elegans*, specifically for defecation cycles [12]–[14]. Some of the genes which act in temperature compensation of this ultradian behavior might be responsible for the modulation of circadian behavior [15].

The finding of a robust circadian behavior in *C. elegans*, which could be recorded automatically [6], [16], would contribute a powerful tool for the study of the biological clock in this model, as well as a screening procedure for the elucidation of its molecular basis. The aim of this study is to fully characterize the circadian rhythm of locomotor activity in adult *C. elegans*, including free-running and entrained conditions, as well as a means for genetic screening in this nematode.

## Results

**Figure 1:**
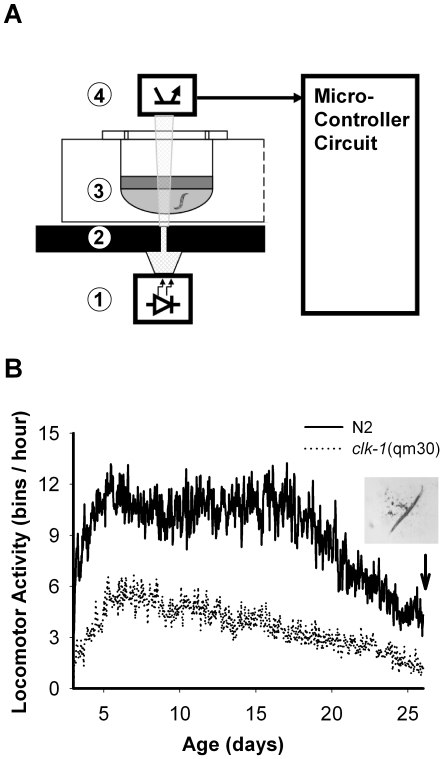
Nematode tracking system. A) Schematic diagram of the tracking system. From bottom to top: 1. IR emitter; 2. Microhole filter; 3. well plate where nematodes are held with 20 µl CeMM + FuDR 40 µM + AB 1x, 20 µl silicon oil (optional to avoid medium evaporation), tape; 4. Phototransistor. The light intensity output is converted to digital format and acquired by a microcontroller system. B) Whole-life recording of locomotor activity in wild-type (N2) and mutant *clk-1*(qm30) (slow moving strain) nematodes. The graph shows average activity of 48 nematodes. FuDR prevented the birth of larvae throughout the experiment (see inset picture).

As we have previously shown, locomotor activity of individual nematodes can be recorded with an infrared microbeam system [6]. In order to increase the number of nematodes to be recorded at the same time, we have modified the system adding a microcontroller to track simultaneously 48 nematodes per plate (figure 1a). This modification plus the optimization of culture conditions allowed us to track locomotor activity for more than 20 days (figure 1b). As a control of the system a slow-moving strain (*clk-1* (qm30) mutant) was recorded, as well as the lethal effects of azide addition (average activity on day 3 of adult stage: N2 (wild type)  = 267 bins/day (SEM = 27, n = 42) ; clk-1 (*qm30*)  = 133 bins/day (SEM = 10, n = 47); N2+azide  = 0 bins/day (SEM = 0, n = 46)) . Our system is then capable of recording locomotor activity corresponding to the probability of the worm interrupting the light microbeam, which correlates with the activity pattern of the animal.

### Locomotor rhythms are entrained by light

When entrained to light-dark cycles, the nematodes showed an activity pattern of 24 hours. However, we observed individual nematodes entrained at different phases with respect to the *zeitgeber* (external time cue); to deal with this problem we conducted population studies described with average plots and phase and frequency histograms.

**Figure 2:**
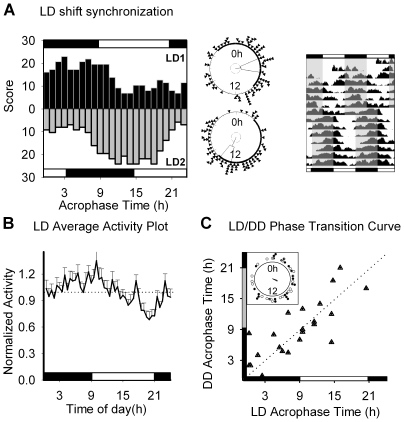
Light synchronization. A) Phase histogram of nematodes entrained to a light-dark cycle (12 h∶12 h 400 lux, 18°C), before and after a 6 h shift of the photoperiod. The middle panel shows the circular statistical analysis (Rayleigh test) for both situations (LD1: lights on at 0900 h; lights off at 2100 h; p<0.05, Rayleigh’s test, n = 80; LD2: lights on at 1500 h, lights off at 0300 h, p<0.05, Rayleigh’s test, n = 81) (the dotted circle inside the plot represents the significance threshold (p = 0.05), to compare with the resultant vector amplitude). The actogram on the right corresponds to average activity of the population. Gray shaded areas represent dark phases of the photoperiod. B) Average daily activity plot corresponding to 3 days of data from 20 nematodes synchronized to a 12 h∶12 h light-dark cycle. The analysis of 3 days of average activity indicates a significant difference between diurnal and nocturnal values (Maximum value  = 1.35±0.10 (10 h); Minimum value  = 0.68±0.09 (20 h); ANOVA test: p<0.0001). c) Comparison of locomotor activity acrophases between LD and the initial days under constant dark (DD) conditions, each triangle corresponds to the mean acrophase (cosinor analysis) of individual nematodes, calculated with the last 3 days of the pre-entrained condition (X axis) vs. the mean acrophase of the 3 first days of the constant dark condition (Y axis). Inset: Rayleigh test showing the phase angle of the population in entrainment (dark circles; r = 0.3 phi = 1.9 (7.3 h) n = 24) and the beginning of the constant condition post entrainment (white circles) (Rayleigh test: r = 0.3 phi = 2.0 (7.6 h) n = 24).

Nematodes were synchronized to white light (400 lux) with a normal distribution of acrophases (peak time), exhibiting higher activity levels around the dark-to-light transition and a minimum at dusk; moreover, nematodes reentrained to the LD cycle after a 6-h shift in the photoperiod (figure 2a). Average activity patterns did not show a clear anticipation to the light or dark phase of the cycle, while overt locomotor behavior appeared to be dampened during the light phase (figure 2b). Since nematodes have been reported to be responsive to light in the 520 to 600 nm bandwidth (green/yellow light) [17], as well as to blue/ultraviolet wavelengths [18], [19], and with the intention to control any possible artifact due to changes in the incubator temperature, we also studied the effect of red light on circadian activity. While circadian rhythms were synchronized by white light – dark cycles (400:0 lux), red light - dark cycles were unable to synchronize activity (Figure S1), suggesting this is a specific effect of white light and is not due to artifactual effects of the stimulus, such as changes in the temperature of the cultures.

When nematodes were released into continuous darkness after 5 days of preentrainment (LD white light 400:0lux), their initial phase in DD could be predicted by the previous phase under entrainment conditions (Figure 2c), suggesting the light cycle was indeed synchronizing the circadian rhythm of locomotion. The correlation between the phase of locomotor activity under LD and DD conditions indicates entrainment; if light were simply masking behavior, a random phase would be expected when animals were placed under constant darkness.

### Locomotor activity rhythms are entrained by temperature cycles

**Figure 3:**
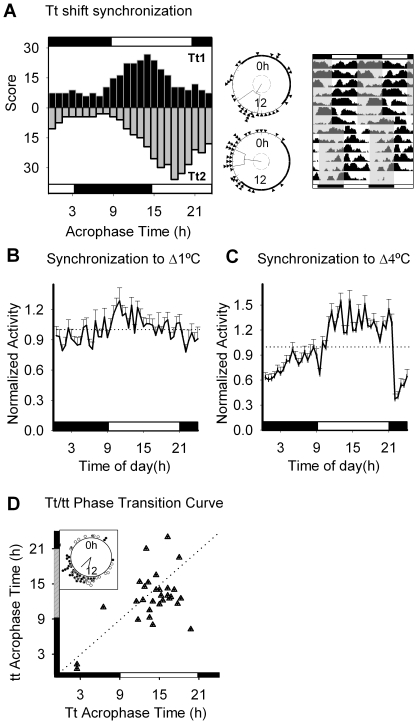
Temperature synchronization. A) Acrophase histogram of nematodes entrained to a high (17°C) - low (16°C) temperature cycle, before and after a 6 h shift of the thermal cycle. The dark-white bar corresponds to cycles of low and high temperature, respectively. The middle panel shows the Rayleigh analysis for both situations: Tt1(17°C from 0900 h to 2100 h, 16°C from 2100 h to 0900 h; p<0.05 n = 32) and Tt2(17°C from 1500 h to 0300 h, 16°C from 0300 h to 1500 h; p<0.05 n = 38). The right actogram corresponds to average activity of the population. Gray shaded areas represent the phase of high temperature of the daily cycle. B) Average daily activity plot corresponding to 3 days of data from 20 nematodes synchronized to a 17∶16°C. There was a significant variation between activity values during the high and low temperature phases (Maximum value = 1.28±0.13 (1100 h) ; Minimum value  = 0.78±0.07 (2200 h), p<0.03, ANOVA). C) Average daily activity plot corresponding to 3 days of data from 20 nematodes synchronized to a 20∶16°C cycle. There was a significant variation between activity values during the high and low temperature phases (Maximum value = 1.55±0.12 (1400 h), Minimum value  = 0.37±0.06 (2200 h); p<0.0001, ANOVA). D) Comparison of locomotor activity acrophases between Tt (20°C∶16°C) and the initial days after release into constant darkness (DD) and constant temperature conditions (16°C). Inset: Rayleigh analysis of locomotor activity acrophase under Tt and tt cycles (Tt (black circles): r = 0.7 phi = −2.4 (14.8 h) n = 35; tt (white circles): r = 0.5 phi = −2.9 (12.9 h) n = 31).

We then considered the role of temperature in synchronizing *C. elegans* circadian rhythms. When nematodes were entrained to cycles of 17°C∶16°C (T∶t 12∶12 h) under constant dark conditions, we observed a normal distribution of acrophases peaking in the beginning / middle of the day, which was reentrained after a 6 h shift in the Tt cycle (considering “day” as the phase with higher temperature; figure 3a). Locomotor activity appeared to be increased during the high temperature phase (figure 3b). Moreover, under a larger temperature variation (20°C∶16°C T∶t 12∶12 h) the amplitude of rhythms was higher and a decrease of activity could be appreciated when temperature went from high to low values (Figure 3c). Also, after a preentrainment stage (5 days Tt 20°C∶16°C T∶t 12∶12 h, DD), when nematodes were subjected to continuous conditions (16°C, DD) their initial phases in DD were determined by those from the entrainment condition (Figure 3d). As in the case of light, this suggests that temperature cycles do indeed entrain circadian locomotor activity rhythms, rather than exert a masking effect on behavior.

### Rhythms are maintained in constant conditions

**Figure 4:**
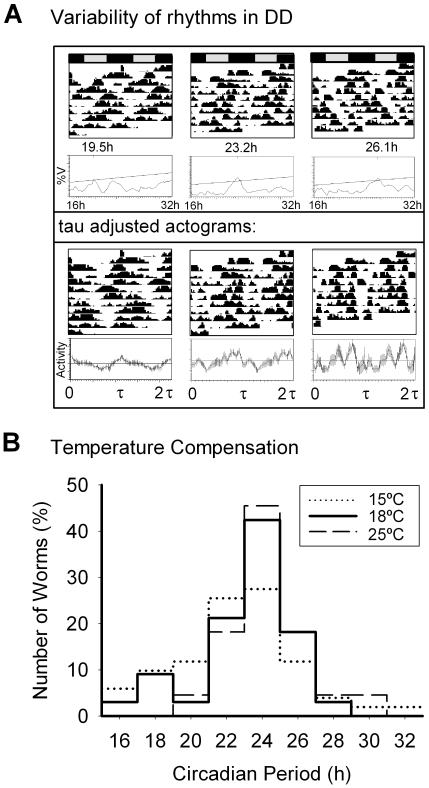
Locomotor activity rhythms in *C. elegans* under constant conditions. A) Examples of individual nematodes exhibiting different circadian periods. Actograms are plotted in 24 h-days (top panel) and replotted in “tau” days (i.e., the length of each cycle corresponds to the circadian period of each animal, see middle panel). The lower panel shows average circadian activity for two cycles. B) Distribution of circadian periods in wild-type *C. elegans* under different temperature conditions.

When studied under constant conditions, individual rhythmicity patterns were partially unstable and a wide range of circadian periodicities was found (figure 4a). Of the total of animals recorded (n>300), 70% were classified as analyzable (in terms of nematodes in good general conditions and high activity levels (average day activity >20% of population mean) at the end of experiment), of which 57% were classified as rhythmic according to the statistical analysis applied (concurrent positive results of chi square and Lomb-Scargle periodograms, as well as visual inspection of the records). Circadian periodicity shows a population distribution of periods centered at 23.9±0.4 h at 18°C. Rhythmic animals showed a normal distribution of circadian periods when recorded at different temperatures ranging from 15°C to 25°C (figure 4b). The population rhythm was found to be temperature compensated with an average Q_10_ = 1.09.

### Circadian mutants

**Figure 5:**
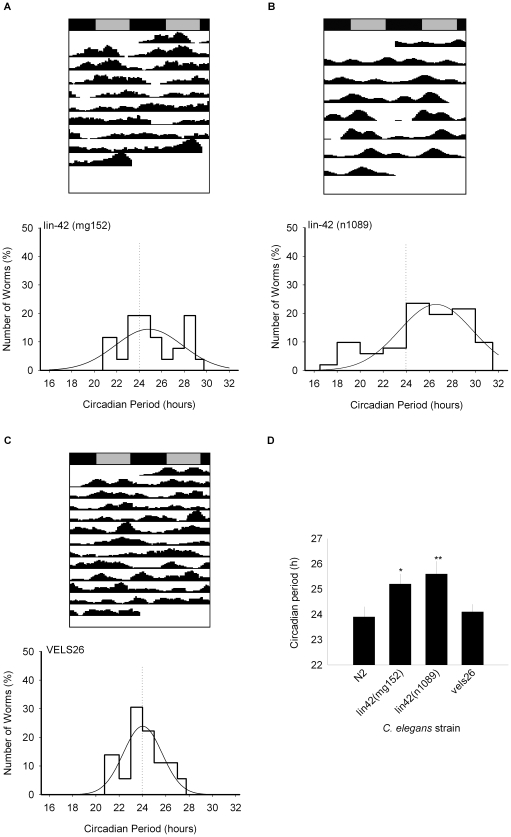
Representative actograms and frequency distribution of circadian periods in *C. elegans*. a) *lin-42*(mg152) mutants (n = 26). b) *lin-42*(n1089) mutants (n = 51). c) Vels26 mutants (which rescue the *lin-42*(mg152) mutation) (n = 36). d) Average circadian periods for control and mutant strains (*p<0.05, **p<0.02 versus N2 controls).

To test whether it is possible to detect mutants with circadian abnormalities, we focused on studying nematode mutants of homologs of other organisms’ clock genes. Interestingly, when we tested the role of *lin-42* (homolog to the clock gene period [9], although apparently not related to adult locomotor behavior) in circadian rhythmicity, we found that mutants of this gene exhibit a longer circadian period. We used two different alleles for this mutation: *lin-42*(n1089), carrying a deletion of 5233 bp which includes the PAS domain of the gene, fundamental for core-clock protein interactions; and *lin-42*(mg152), which has an 8 bp deletion, a 2 bp insertion and a stop cordon, where the PAS domain is also lost. Both mutants have been recently related to developmental abnormalities [20]. *Lin-42*(mg152) mutants had an average circadian period of 25.2±0.4 h (and their period distribution was significantly different from N2 controls; p<0.05), which is rescued to 24.1±0.3 h in the transgenic strain VELS26 (lin-42::gfp, p>0.05 versus N2 controls) (Fig. 5). *lin-42*(n1089) mutants also exhibited a significant increase in circadian period (25.6±0.5 h, p<0.02 versus N2 controls). Lin-42 mutants were entrained to light and temperature cycles in a similar way as controls (data not shown).

We have also tested mutants of *aha-1*, homolog to the *bmal* gene. Since null mutations for *aha-1* are lethal [21], we have studied a rescue mutant strain (*2g8*) in which the gene is expressed in non-neural areas of the pharyngeal bomb [22]. No significant differences between this strain and control nematodes were found in terms of circadian period (24.2 h) or entrainment to light or temperature cycles (data not shown).

## Discussion

Our results represent a complete description of the locomotor activity rhythm in *C. elegans*, with a methodology that allowed us to uncover three of the key features of circadian systems: entrainment, free-running and temperature compensation. In addition, we have found a role for a gene homolog to the clock gene *period*, well-known for its participation in the molecular clock in several groups including mammals and flies.

Although we have succeeded in recording locomotor activity rhythms in individual nematodes, the high variability between periods and acrophases is indeed a challenge and supports the need of populational studies for screening purposes of circadian clock mutants. With a standard deviation of 3.0 h, the minimum number of rhythmic nematodes to detect a significant difference between two populations (with 2 hours difference in average) would be 36 [23]. However, taking into account the proportion of rhythmic animals in the records, this number might be increased to more than 60. Unpublished observations from other groups using different methods for recording locomotor activity (i.e., videotracking) support our observation of a high variability in *C. elegans* circadian parameters, as well as an important proportion of arrhythmic individuals (Hut & Merrow, University of Groningen, personal communication), in contrast with previously published data [3] which suggested a homogeneous distribution of circadian rhythms in this model. Although smaller variations in the parameters of hermaphroditic animals could be expected, it could be attributed to the structure of the timekeeper itself. Moreover, our data resemble stochastic clock models that use a small number of components in their calculations [24], [25].

Nematode circadian rhythms have been recorded with two different methods. Video tracking, originally described by Saigusa et al. [3] allows the recording of individual nematodes with a “bug tracker”; however, as discussed previously [6] it is not particularly useful for mutant screening and circadian population characterization. An alternative would be to use a “parallel worm tracker” for this type of studies, as described by Ramot et al. [26], which has not been tested in long-term circadian experiments. We have previously demonstrated that our system is particularly useful for long-term recording of circadian rhythms [6]. In addition, circadian rhythms were demonstrated by testing circadian tolerance to stressful stimuli every 4 hours in aliquots from a nematode larvae liquid culture [4]. This method is extremely time-consuming and probably not applicable for mutant screenings, and although we have replicated a similar technique with adults [5], it seems to work better with larvae. The present work is focused on the characterization of adult locomotor behavior and synchronization by light or temperature, so we opted for our own technical approach.

In addition, it is important to state that a populational approach has been very successful for the determination of key features of the circadian system. Indeed, the classical studies of *Drosophila* eclosion rhythms by Colin Pittendrigh [27], as well as the first description of circadian mutations in flies [28] relied on a large number of individuals which allowed to characterize the rhythms in the population.

Circadian rhythms in *C. elegans* might respond to a canonical molecular oscillator as the one described in *Drosophila* and mammals [1]. However, although several genes with diverse degrees of homology to clock genes have been described in this species [7], they might serve other functions in nematodes [9], [10], [20]. Indeed, we have tested circadian rhythmicity in two lin-42 (a homolog of the clock gene *period*) mutants, mainly expressed in developmental stage transitions in *C. elegans* [9] and found a significant increase in circadian period as compared to wild type controls. Moreover, a genetic rescue strain of this mutation, transgenic nematodes VELS26 (lin-42::gfp), that restores the heterochronic phenotypes observed in lin-42(mg152) [9], also recover a normal circadian period. Therefore, we suggest that a molecular pacemaker sharing at least some of the key elements as those found in other systems might be present in *C. elegans*. Although the molecular machinery sustaining circadian rhythms in nematodes might be somewhat different from other, well-known models, it is also plausible that a different mechanism guides *C. elegans* cyclic behavior.

It is tempting to speculate about the role of circadian rhythms in an underground organism. *C. elegans* was originally isolated from several habitats, including compost, mushroom beds, garden soil and water, as well as associated with several other species [29]. In this sense, a circadian system might render the species more adaptive to a cyclic environment, even if temporal signals are relatively weak in the original habitat. Although the environmental time cue (*zeitgeber*) responsible for entrainment in this species in the wild is unknown, in this work we have demonstrated a synchronizing role for both photic and temperature signals. *C. elegans* is photoresponsive to visible light (540 nm) to intensities as low as 40 lux [17], although the photoreceptive mechanism is not well understood. Circadian rhythms are found in organisms living under extreme conditions [30], [31], so the problem of sensing an environment which gives weak cyclic cues is definitely relevant for survival.

An additional mechanism for photodetection has been recently described in this species. Although *C. elegans* lacks the canonical photoreceptor cells or pigments, it can behaviorally respond to blue and/or ultraviolet light detected by specific receptors of the invertebrate Gustatory receptor (Gr) family. Moreover, negative phototaxis has been shown to depend on the activity of cGMP-sensitive cyclic nucleotide-gated (CNG) channels or cAMP-related invertebrate gustatory receptors [18], [19]. It is tempting to speculate that these photoreceptor systems might also be involved in circadian entrainment in this species; indeed, more experiments including a complete spectral analysis for light responses should be performed. In addition, G-protein coupled receptors have been implicated in a variety of sensory capabilities in *C. elegans* (e.g., [32]) and should be considered as putative candidates mediating signal transduction pathways related to circadian entrainment in this species.

Our data suggests that temperature is also a strong signal for circadian setting, since low amplitude cycles (i.e., 1°C) are able to entrain locomotor activity cycles. Although it is possible that white light cycles also include a thermal component, the fact that in our experiments other wavelengths failed to entrain rhythms suggests that photic signalling is able to affect oscillations by itself. Indeed, both *zeitgebers* might be important for entrainment, and the circadian clock responsible for locomotion cycles could integrate both signals at a molecular level, as has been recently shown in Drosophila [33], [34]. Finally, it is possible that other Zeitgebers are effective in the wild, including chemical gradients or even neurohumoral exocrine communication (i.e. pheromones or even melatonin [35], [36]). It is noteworthy that entrainment to light or temperature cycles yields different results in terms of the peak in locomotion levels, suggesting that different mechanisms might be responsible for synchronization to diverse environmental cues. Indeed, *zeitgeber* competition experiments might be useful to determine the relative weight of each stimulus in entrainment mechanisms.

The initial characterization of the *per* gene in *Drosophila* consisted of arrhythmic, short-period and long-period phenotypes [28] and eventually led to the transcription-translation model of the molecular circadian clock, which is supported by mutations in the *per* gene (and others) in mammalian models [37]. Indeed, most eukaryotic circadian systems encode a PAS-containing positive transcription factor consisting in Clock/Cycle (or Bmal in mammals) interacting with the negative regulator Per. Since mammals and flies share this mechanism, it can be stated that this molecular circadian pacemaker evolved from a common ancestor about 500 million years ago, but might have a bi-phyletic origin since bacterial pacemaker genes have no relation with animal counterparts [38]. Our initial screening method suggests a role for lin-42, homolog to the mammalian and Drosophila *per* gene [9], [20], in the determination of circadian periodicity in *C. elegans*. The finding of a role for a *per* homolog in *C. elegans*, together with the search for other circadian homologs in this species [7] strengthens the view of a common circadian mechanism in the animal kingdom. Among these putative homologs there are a number of kinases worth studying, in particular casein kinases, which have been shown to be key post-translational regulators of the circadian loop, in particular through its modulation of *per* kinetics [39]. The exact mechanism through which *lin-42* affects the circadian machinery in this species remains to be determined.

In conclusion, we have shown that locomotor activity rhythms in *C. elegans* are generated by an endogenous pacemaker and are sustained under constant environmental conditions. Such behavior is temperature compensated, a feature that has been found for most circadian behaviors. Light and temperature are adequate entrainment agents and also increase the amplitude of these rhythms. However, individual records are often unstable and exhibit a high degree of variability, both in terms of period (under constant conditions) and phase of entrainment under different *zeitgebers*. Under laboratory conditions, we suggest that a populational approach is more adequate for the study of circadian rhythmicity in *C. elegans*, which could allow for a screening system for chronobiological mutations and would be useful for the study of the elusive clock of this model system. In this aspect, our finding of a *lin-42* circadian phenotype puts *C. elegans* in the circadian map and encourages the use of this notable model system in chronobiological studies.

## Materials and Methods

### Strains and culture conditions

Wild-type *C. elegans* N2, *clk-1*(qm30) and *lin-42*(n1089) mutants were provided by the Caenorhabditis Genetic Center (Minneapolis). The *lin-42*(mg152) and the VELS26 (lin-42::gfp) strain were gifts from Ann Rougvie (University of Minnesota).

Nematodes were cultured in NGM medium with thick bacterial lawns of *E. coli* OP50 strain [40]. Stock plates were maintained and grown at 20°C. At L4 stage, one hermaphrodite was picked to a well of a 96 microtiter plate with 50 µl of CeMM liquid medium [41] (Cellgro, generously donated by N.J. Szewczyk, University of Pittsburgh), plus 40 µM fluorodeoxyuridine (FUdR) to prevent self-reproduction and 1X Antibiotic/Antimicotic (AB) (Gibco, Rockville, MD). The well was sealed with 30 µl of silicone oil plus tape to prevent desiccation and 2 small holes were perforated in the border with a needle to allow for oxygen interchange and to prevent water condensation (Kippert, F., personal communication).

### Experimental conditions

For light synchronization experiments, nematodes were subjected to light:dark cycles (400:0 lux 12∶12 h) under constant temperature (18°C). Stability of the incubator temperature was checked with an IButton sensor model DS1921H-F5 (Maxim Integrated Products, Inc. Sunnyvale, CA), programmed to take 1 sample every 5 minutes. This sensor provides a resolution of 0.125°C. There was no temperature variation in the recording chamber throughout the experiments (within the resolution of the temperature sensor).

The light source employed throughout the experiments was a Compact Fluorescent Lamp Philips Essential PLE15W230 (the standard fluorescent lamp spectrum can be found in http://www.geciasia.com/apo/resources/library/lighting/prod_catalogues/ downloads/CompactFluorescent.pdf), and it is interesting to note that the spectrum peaks in the green and blue zones, coincident with what has been reported for *C. elegans*’ photic sensitivity [17], [19].

For temperature synchronization experiments, nematodes were subjected to High:Low (T∶t) temperature cycles (17∶16°C or 20∶16°C 12∶12 h) under constant darkness.

For experiments in constant conditions, nematodes where kept at a constant temperature (15°C, 18°C or 25°C) under constant darkness.

### Data acquisition and analysis

Data was sampled at 1-min intervals and binned in 30-min blocks. The raw data was detrended and normalized by dividing each data point by the corresponding point of a trend curve (fitted by a 72 h low-pass Butterworth filter), as described [42]. The Butterworth filter, similar to a low- and high-pass filtering with moving average, is widely applied in noisy data (such as the analysis of fly courtship songs) or for the analysis of locomotor activity of flies in order to remove the high frequency components and avoid possible artifacts in peak detection [42], [43]. High frequencies were removed by a 4 h moving average window. Animals with very low activity levels (<20% of average population activity) were discarded.

For synchronization experiments, the filtered data corresponding to at least 5 days of entrainment condition was analyzed by Cosinor analysis (best fit to a 24 h cosine waveform). Nematodes presenting a statistically significant rhythm were used for this analysis and actograms were constructed (with the scale adjusted according to the lower threshold) for better visualization of rhythms. The acrophases (time at peak) for each individual worm were scored and grouped for histogram plotting.

The statistical significance of population entrainment was tested by circular statistics (Rayleigh test [44]). For this analysis, each individual worm was analyzed by Cosinor and the mean acrophase was employed in the test. The significant threshold (dotted circle in the plots) was set to p = 0.05. Cosinor analysis yields a confidence interval for acrophases, and we have assigned scores of “1” to all acrophase values within the interval. Scores were integrated for all animals and graphed as histograms, which are more representative of the population than a standard percentage histogram based on the mean acrophase for each animal.

Average activity plots were constructed with 3 days of locomotor activity data, using the individual detrended raw data normalized by the mean daily value (adapted from the *Drosophila’s* IandA Excel macro).

In order to compare the phase of rhythms under entrained (by light or temperature) and free running conditions, we constructed phase transition (PTC) plots [45], by using the mean acrophase value of cosinor analysis for the last 3 days of the entrainment condition and the first 3 days in constant darkness and temperature conditions.

For DD experiments, the filtered data was plotted and classified in terms of high amplitude (>1.5x between subjective day and night) and stable rhythmicity. These preselected nematodes were analyzed by chi^2^ and LS periodograms and the periods of animals with statistically significant circadian rhythms were plotted as a frequency histogram. When important differences were found between both type of periodograms or between statistical analysis and visual inspection, the worm was discarded from further analysis. Histograms were adjusted to a Gaussian curve using the Sigmaplot software. The median and variances of period distribution were analyzed by T-test for samples with unequal variances and F-test, respectively.

In all cases, data are presented as mean ± standard error.

## Supporting Information

Figure S1 Red light does not entrain C. elegans locomotor activity rhythms. a) Distribution of acrophases in animals subjected to white light-dark and red light-dark cycles (left panel). Rayleigh analysis of acrophases under both conditions (right panel) White light entrainment: p<0.05, Rayleigh test, n = 32; Red light entrainment: p>0.05, Rayleigh test, n = 27. b) Average plot for daily activity under both conditions (white light entrainment: p<0.004, n = 20, ANOVA; red light entrainment: p>0.05, ANOVA, n = 20).
(0.46 MB TIF)
